# Chronic Diarrhea and Pancolitis Caused by Paracoccidioidomycosis: A Case Report

**DOI:** 10.1155/2010/140505

**Published:** 2010-06-30

**Authors:** Eduar A. Bravo, Arturo J. Zegarra, Alejandro Piscoya, José L. Pinto, Raúl E. de los Rios, Ricardo A. Prochazka, Jorge L. Huerta-Mercado, Nancy L. Mayo, Martin Tagle

**Affiliations:** ^1^Deparment of Gastroenterology, Hospital Nacional Cayetano Heredia, Lima 31, Peru; ^2^Deparment of Pathology, Hospital Nacional Cayetano Heredia, Lima 32, Peru; ^3^Deparment of Gastroenterology, Clinica Anglo-Americana, Lima 18, Peru

## Abstract

South American blastomycosis is a systemic micosis caused by infection with Paracoccidioides brasiliensis. The most frequently affected sites are the lower lip buccal mucous membrane, palate, tongue, sublingual region, lymph glands, and lungs. However, colonic involvement is not a common expression of Paracoccidioidomycosis. We report a case of chronic diarrhea and pancolitis caused by Paracoccidioidomycosis with fatal outcome.

## 1. Introduction

South American blastomycosis is a systemic micosis caused by infection with Paracoccidioides brasiliensis [1], a dimorphic fungus, and is endemic in humid tropical and subtropical zones of continental Latin America, Brazil, Perú, and Colombia [2]. The first infection usually occurs in the first 2 decades of life through inhalation of the conidia of the mold into the alveoli. The organisms change to the yeast form in the lungs and then multiply through budding and hematogenous dissemination occurs without clinical manifestations but it can also develop many years later, depending on multiple factors involved in the host's immune response (age, use of immunosuppressive drugs, concurrent diseases, etc.) [3–5].

In the classical form of the disease, the most frequently affected sites are the lower lip buccal mucous membrane, palate, tongue, sublingual region, lymph glands, and lungs associated with fever, asthenia, and weight loss [6]. Although adrenal, bone, pancreas, urogenital, spleen, and liver disease are known, colonic involvement is not a common expression of Paracoccidioidomycosis [7].

We report a case of chronic diarrhea and pancolitis caused by Paracoccidioidomycosis.

## 2. Case Report

A thirty-nine-year old female patient, from the state of Chanchamayo, Junin, Peru, with a medical history of recurrent infections of *Strongyloides stercoralis,* came to our hospital with four months of daily bloody mucous diarrhea associated with abdominal pain and 10 kilogram weight loss. Diarrhea episodes increased before admission and she also had mild abdominal colicky pain and fever. Physical examination revealed a wasted patient with pale skin and tenderness at the lower abdominal region. There was no hepatomegaly, splenomegaly, nor palpable lymph nodes. Relevant blood tests showed anemia (Hemoglobin 76 g/L) low albumin (16 g/L). She had over 100 leucocytes and red blood cells per field at the stool examination. Parasitological and stool culture studies were negatives. 

**Figure 1:**
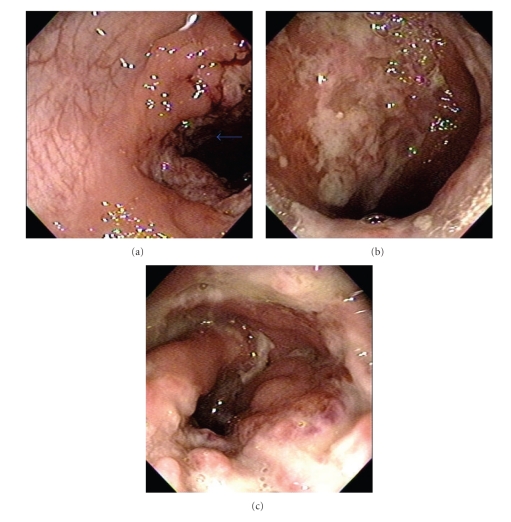
(a) Arrow showing patchy ulcer surround by normal mucosa. (b) Giant exudative colonic ulcer. (c) Intense inflammatory stenotic ulcer.

**Figure 2:**
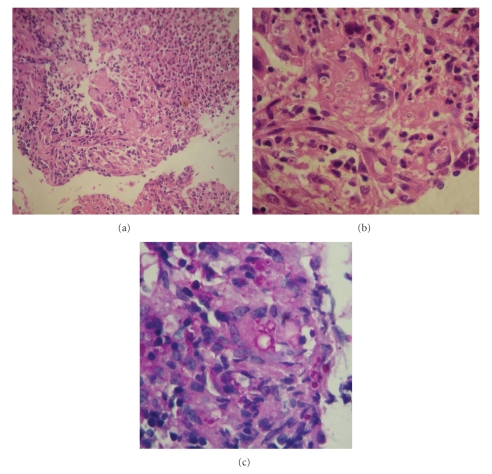
(a) H&E Granulomatous infiltration and necrosis. (b) H&E Giant cell containing multiples PCM. (c) PAS Intra and extracellular PCM.

We found incipient lower infiltrates at chest X-ray and Elisa-HIV was negative. Colonoscopy revealed multiple patchy exudative ulcers from rectum to cecum together with normal mucosa (Figure 1). Biopsies were taken from the edges of the ulcerations. Histopathology shown multiple granulomas and Paracoccidioidomycosis associated with active inflammation (Figure 2).

Sputum and urine samples for Paracoccidioidomycosis and blood test for HTLV-1 were positive. The patient received Amphotericin B as initial treatment with partial response but developed a stroke and died from sepsis.

## 3. Discussion

Paracoccidioidomycosis (PCM) or South American blastomycosis is a granulomatous, chronic, infectious, subacute, or seldom acute disease, caused by the fungus *Paracoccidioides brasiliensis* [2]. It is characterized by a polymorphism of lesions and can affect any organ, but it is usually found in the skin, lungs; the oral and nasal mucous membranes [3, 4].

Eighty to ninety percent of affected individuals are men between 29 and 40 years old, predominantly rural workers, with a male/female ratio of 15  :  1 [4]. The biggest risk factors for acquisition of the infection are activities related to the handling of soil contaminated by the fungus, as in agricultural activities, earth moving, gardening, and transport of vegetable products.

Intestinal involvement is mostly through hematogenous spread but it may occur by ingestion. Clinical spectrum range from abdominal pain, constipation, diarrhea to acute abdomen secondary to appendicitis or intestinal occlusion as a result of the increase in lymph nodes [8, 9].

The disease can present with long latency periods and some no autochthonous cases can even develop more than 30 years after the individual has left the endemic area. Moreover, intestinal form has and incidence of 2.7% to 28.4% of autopsies [10].

The main alterations in the intestinal tract are found in the small and large bowel, in segments rich in lymphoid tissue, such as terminal ileum, appendix, and right hemicolon [8].

Although the intestinal form has nonspecific radiographic aspects, abdominal lymph node calcifications and annular stenosis are suggestive of the disease [8]. Colitis and colonic ulcers are difficult to differentiate from other fungal infection, tuberculosis, inflammatory bowel disease, and even colon cancer [7, 8].

Diagnosis is suspected on clinical grounds, epidemiologic history, and imaging studies and confirmed by the identification of the fungus by culture, direct mycologic, or histopathologic examination [3].

Typical histology shows granulomas rich in epithelioid and giant cells, some containing variable amounts of parasites. The finding of a double wall parasite with simple or multiple gemmulation is diagnostic [3, 4].

PCM treatment includes the use of antifungal drugs, nutritional support, treatment of the eventual sequelae and comorbidities, and the prevention of opportunist diseases [11].

Before antifungal drugs were available, pharmacologic treatment was based on sulfonamides (sulfamethoxazole-trimethoprim) [7]. Currently amphotericin B (AmB), and imidazolic derivates, such as, itraconazole, and fluconazole are the preferred agents [4, 11].

According to the latest guidelines [11, 12]. Patients with severe disseminated disease should be treated with AmB deoxycholate or a lipid formulation of AmB (total dose greater than 30 mg/kg) and mild to moderate disseminated blastomycosis that does not involve the CNS should be treated with itraconazole (200 or 400 mg orally daily).

No consensus exists on the duration of treatment and criteria for its termination have not been established [4]. Recurrences are not uncommon, especially in cases of treatment abandonment. If clinical manifestations reappear or fungal structures of *P. brasiliensis* are isolated, a new treatment course should be undertaken [4].

## References

[1] Al Doory Y, Pairon R (1975). A bibliography of blastomycosis and paracoccidioidomycosis. *Mycopathologia*.

[2] Mackinnon JE (1972). Geographical distribution and prevalence of paracoccidioidomycosis. *Paracoccidioidomycosis: Proceedings of the 1st Pan American Symposium*.

[3] Marques SA (1998). Paracoccidioidomicose. *Anais Brasileiros de Dermatologia*.

[4] Ramos-e-Silva M, Saraiva LDES (2008). Paracoccidioidomycosis. *Dermatologic Clinics*.

[5] Pang KR, Wu JJ, Huang DB, Tyring SK (2004). Subcutaneous fungal infections. *Dermatologic Therapy*.

[6] Restrepo A, Robledo M, Giraldo R (1976). The gamut of paracoccidioidomycosis. *American Journal of Medicine*.

[7] Penna FJ (1979). Blastomycosis of the colon resembling clinically ulcerative colitis. *Gut*.

[8] Chojniak R, Vieira RA, Lopes A, Silva JC, Godoy CE (2000). Intestinal paracoccidioidomycosis simulating colon cancer. *Revista da Sociedade Brasileira de Medicina Tropical*.

[9] Muñoz  A, Chaparro E, Ferrufino J, Vasquez L (2006). Apendicitis caused by *Paracoccidioides brasilensis*. *Revista Medica Herediana*.

[10] Montenegro MR, Franco M, Franco M, Lacaz CS, Restrepo-Moreno A, Del Negro G (1994). Pathology. *Paracoccidioidomycosis*.

[11] (2005). The IX international meeting on paracoccidioidomycosis, Aguas de Lindóia, SP, Brazil. *Revista do Instituto de Medicina Tropical de São Paulo*.

[12] Chapman SW, Dismukes WE, Proia LA (2008). Clinical practice guidelines for the management of blastomycosis: 2008 update by the infectious diseases society of America. *Clinical Infectious Diseases*.

